# Male fertility status is associated with DNA methylation signatures in sperm and transcriptomic profiles of bovine preimplantation embryos

**DOI:** 10.1186/s12864-017-3673-y

**Published:** 2017-04-05

**Authors:** Jenna Kropp, José A. Carrillo, Hadjer Namous, Alyssa Daniels, Sana M. Salih, Jiuzhou Song, Hasan Khatib

**Affiliations:** 1 Department of Animal Sciences, University of Wisconsin-Madison, Madison, WI 53706 USA; 2 Department of Animal and Avian Sciences, University of Maryland, College Park, MD 20742 USA; 3 Department of Obstetrics and Gynecology, University of Wisconsin-Madison, Madison, WI 53792 USA; 4 Present address: Department of Obstetrics and Gynecology, West Virginia University, Morgantown, WV 26508 USA

**Keywords:** Fertility, Embryonic development, Methylation, Transcriptome, IVF

## Abstract

**Background:**

Infertility in dairy cattle is a concern where reduced fertilization rates and high embryonic loss are contributing factors. Studies of the paternal contribution to reproductive performance are limited. However, recent discoveries have shown that, in addition to DNA, sperm delivers transcription factors and epigenetic components that are required for fertilization and proper embryonic development. Hence, characterization of the paternal contribution at the time of fertilization is warranted. We hypothesized that sire fertility is associated with differences in DNA methylation patterns in sperm and that the embryonic transcriptomic profiles are influenced by the fertility status of the bull. Embryos were generated in vitro by fertilization with either a high or low fertility Holstein bull. Blastocysts derived from each high and low fertility bulls were evaluated for morphology, development, and transcriptomic analysis using RNA-Sequencing. Additionally, DNA methylation signatures of sperm from high and low fertility sires were characterized by performing whole-genome DNA methylation binding domain sequencing.

**Results:**

Embryo morphology and developmental capacity did not differ between embryos generated from either a high or low fertility bull. However, RNA-Sequencing revealed 98 genes to be differentially expressed at a false discovery rate < 1%. A total of 65 genes were upregulated in high fertility bull derived embryos, and 33 genes were upregulated in low fertility derived embryos. Expression of the genes *CYCS, EEA1, SLC16A7, MEPCE,* and *TFB2M* was validated in three new pairs of biological replicates of embryos. The role of the differentially expressed gene *TFB2M* in embryonic development was further assessed through expression knockdown at the zygotic stage, which resulted in decreased development to the blastocyst stage. Assessment of the epigenetic signature of spermatozoa between high and low fertility bulls revealed 76 differentially methylated regions.

**Conclusions:**

Despite similar morphology and development to the blastocyst stage, preimplantation embryos derived from high and low fertility bulls displayed significant transcriptomic differences. The relationship between the paternal contribution and the embryonic transcriptome is unclear, although differences in methylated regions were identified which could influence the reprogramming of the early embryo. Further characterization of paternal factors delivered to the oocyte could lead to the identification of biomarkers for better selection of sires to improve reproductive efficiency.

**Electronic supplementary material:**

The online version of this article (doi:10.1186/s12864-017-3673-y) contains supplementary material, which is available to authorized users.

## Background

Mammalian infertility is of concern to both human couples seeking to establish a family and also in the dairy industry to meet production demand. In couples seeking assisted reproductive technology, male infertility accounts for 40% of all couples’ infertility diagnosis [1], and it is estimated that genetic abnormalities are present in about 15% of infertile males [2]. Likewise in dairy cattle, infertility is a multifactorial problem where reduced fertilization rates, low conception rates and a higher degree of embryonic mortality have become challenges to improving dairy cattle reproductive efficiency [3–6]. Though infertility is a complex trait, the study of the genetic component of sperm is advantageous as it could be easily screened for biomarkers of fertility and moreover, the paternal influence on subsequent embryonic development is relatively unexplored.

The genetic contribution of sperm in relation to fertility has been of recent interest across mammalian species. It is now well understood that, at the time of fertilization, the spermatozoa delivers more than just paternal DNA, but rather an entire package including RNAs, transcription factors, and cell signaling molecules [7]. Indeed, a study by Ostermeier et al. [8] was the first to show through zona-free hamster egg/human sperm penetration tests that not only were RNAs delivered by sperm, but also were proposed to have roles in the early zygote. Card et al. [9] profiled the transcriptome of bull spermatozoa and identified 6166 transcripts in which about 66% were full-length transcripts. Transcripts detected within spermatozoa in the study included *PLCZ1* and *CRISP2*, both of which have roles in fertilization. The authors concluded that full-length transcripts within transcriptionally inactive sperm could plausibly be translated after spermatogenesis to have roles in the early development of the embryo.

Several studies have sought to characterize the differences in sperm RNA between males of differing fertility [10–15]. In humans, a microarray study detected 5382 transcripts in which 157 transcripts were up- or down-regulated in sperm of oligozoospermic infertile men compared to fertile men [12]. The differentially expressed transcripts were of genes with roles in spermatogenesis, DNA repair, oxidative stress, and histone modifications. Similarly, the transcriptome of sperm has been characterized for bulls of differing fertility. For example, studies assessed the mRNA expression of proteins associated with sperm function in bulls of differing sires conception rate (SCR) and found several genes correlated with either high or low fertility bulls [10, 11]. Moreover, a more comprehensive microarray analysis study identified 415 transcripts to be differentially expressed between high and low fertility bulls, where the population of transcripts in low fertility sperm was deficient in transcriptional and translational factors [15]. Collectively, these studies suggest that the transcriptome is drastically different between sires of high and low fertility, and the presence of certain transcripts is associated with infertility. Although several transcripts are associated with fertility status, the effect of the delivery of these transcripts at fertilization to the oocyte and their roles in early embryonic development is not well understood.

Sire fertility has been evaluated in terms of physical quality parameters, including motility and morphology as well as the RNA profile associated with a given fertility index. While the sperm transcriptome has been characterized across sire fertility indices, previous studies have not determined whether the embryonic transcriptome is influenced by the “RNA package” delivered by sires of differing fertility status at the time of fertilization. We hypothesized that bulls of differing fertility will have different DNA methylation signatures and will affect not only the development of the early preimplantation embryo, but also the transcriptome of the embryo. Here, we first aimed to assess whether embryo morphology and development, in terms of fertilization and blastocyst rate, differed between embryos derived from high and low fertility sires. The second aim was to characterize the embryonic transcriptome of embryos derived from high and low fertility sires to determine whether the sire’s fertility has a genetic effect on the embryo and to potentially identify differentially expressed genes. The third aim was to characterize DNA methylation signatures of bulls differing in their fertility status. Utilizing an in vitro fertilization (IVF) system, embryos were generated from either a high or low fertility sire allowing for the analysis of the paternal influence on the embryonic transcriptome. Understanding how the preimplantation embryonic transcriptome may be impacted by paternal factors could facilitate the identification of paternal RNAs, microRNAs and transcription factors that drive embryonic development. These factors attributed to the paternal genome may be implicated in “differential fetal programming”, and could serve as biomarkers of bull fertility.

## Methods

### Bull selection

Sires were chosen based on extreme SCR which is defined as a percent increase or decrease in conception rate for a given sire relative to the herd’s average. The SCR is an evaluation performed on bulls with greater than 300 mating records within the last 4 years across a minimum of 10 herds (https://aipl.arsusda.gov/reference/arr-scr1.htm). Semen from 12 bulls was donated by Genex Cooperative, Inc., where six bulls were deemed as high fertility bulls and six were deemed as low fertility bulls. The bulls selected represent the extreme sires for the SCR measure within the company’s marketed Holstein sire pool. The measure of SCR and corresponding percent accuracies were as follows: high fertility sires were 5 (97%), 4.1 (93%), 3.9 (95%), 3.8 (95%), 3.7 (97%), 3.4 (99%) whereas the low fertility sires were -2.3 (81%), -2.7 (72%), -3.8 (94%), -3.9 (86%), -5.3 (78%), -7.5 (90%).

### In vitro production of embryos

IVF experiments were previously described by Khatib et al. [16] and Driver et al. [17], and here are described in brief. Ovaries were purchased from Applied Reproductive Technology, LLC (Monona, WI) and transported in saline solution held at 39° Celsius. These ovaries were obtained from a slaughterhouse where the majority of cows processed at the time of collection were of the Holstein breed. The antral follicles were aspirated for cumulus-oocyte complexes (COCs). To minimize a dam effect, recovered COCs from all ovaries were pooled together for each experiment. The COCs were then washed in Tyrode's albumin lactate pyruvate (TALP)-Hepes medium and transferred in groups of 10 into a 50 μl drop of M-199 medium supplemented with gonadotropins (3 μg/ml each of FSH and LH) estradiol, 25 μg/ml of gentamicin sulfate, 0.22 mM sodium pyruvate, and 10% fetal bovine serum. The COCs were then incubated at 39 ° C, 95% humidity and 5% carbon dioxide for 24 h.

Following oocyte maturation, groups of 10 COCs were washed once in TALP-Hepes. Each cohort was placed into a 44 μl drop of fertilization medium consisting of IVF-TL (Specialty Media, Phillipsburg, NJ) supplemented with 0.22 mM sodium pyruvate, 25 μg/mL gentamicin sulfate and 6 mg/ml essentially fatty acid-free bovine serum albumin (FAF-BSA; Sigma-Aldrich, Catalog No. A-8806). It is important to note that prior to fertilization, COCs from each maturation culture plate were divided between two fertilization culture plates in order to randomize the oocyte population prior to fertilization. These fertilization plates were then fertilized with frozen-thawed semen by either a high fertility or low SCR bull, where a total of 150–350 oocytes per bull were fertilized per IVF replicate. Semen was prepared using a Percoll discontinuous gradient as described by Parrish et al. [18], and adjusted to a final concentration of one million/ml. Oocytes were co-cultured with sperm (day 0) in fertilization medium supplemented with heparin and PHE. Once fertilized, the presumptive zygotes were incubated for 20 h. Following incubation, the zygotes were stripped of their cumulus cells, washed once in TALP-Hepes medium and placed in groups of 25 per 50 μl drop of SOF medium (Specialty Media) supplemented with 0.22 mM sodium pyruvate, 25 μg/ml gentamicin sulfate, and 8 mg/ml FAF-BSA. Embryos were assessed on day 8 of culture for blastocyst stage and quality. A total of two biological IVF replicates were carried out per high/low SCR bull pair, where a pool of morphologically similar expanded blastocysts derived from each high and low SCR bull was collected, respectively, per IVF replicate. According to the embryo evaluation criteria described by Bo and Mapletoft [19], blastocysts of stage 7 and quality grades 1 and 2 were collected for each bull. Embryos within pools generated from three high/low bull pairings were utilized for RNA-Seq analysis and three additional high/low bull pairings were utilized for validation of RNA-Seq results by qRT-PCR.

Statistical analysis of development data was performed in the program R (www.r-project.org/) using mixed models taking into account the IVF replicate and bull effect. The significance of the bull effect was tested using a likelihood ratio test comparing the full model against a model without the treatment effect, analyzing the response variables fertilization and blastocyst rates.

### Extraction of RNA from embryos and RNA amplification

Total RNA was extracted from each pool of blastocysts (*n* = 46–63) using the RNaqueous Micro-Kit (Life Technologies, Grand Island, NY) and then underwent one round of linear amplification using the MessageAmp II aRNA amplification kit (Life Technologies). Samples were quantified and quality checked using a Qubit® 2.0 Fluorometer (Life Technologies) and Agilent 2100 Bioanalyzer (Agilent, Santa Clara, CA), respectively.

### Library preparation and RNA-Sequencing

Equal amounts of RNA were used to prepare cDNA libraries using the ScriptSeq™ v2 RNA-Seq Library Preparation kit (Epicentre, Madison, WI) following the recommended protocol for the kit. Libraries of cDNA were then quantified and quality checked using a Qubit® 2.0 Fluorometer (Life Technologies) and Agilent 2100 Bioanalyzer (Agilent), respectively. Libraries were then sequenced using an Illumina HiSeq 2000 at the University of Wisconsin-Madison Biotechnology Center.

### RNA-Sequencing data analysis

Data analysis was performed by the University of Wisconsin-Madison Biotechnology Center. Sequencing reads were trimmed to remove sequencing adaptors and low-quality bases and were then aligned to the bovine reference genome UMD 3.1 utilizing the default parameters of the alignment software STAR v2.4.0j [20]. Quantification of expression for each gene was calculated by RSEM v1.2.16 utilizing the default parameters, where both transcripts per million reads (TPM) and expected read count were computed [21]. The expected read counts were used for differential expression analysis using EBSeq v1.1.5 [22], using the RSEM package and a false discovery rate (FDR) of 0.05.

### Gene expression validation by real-time quantitative PCR

To confirm the differential expression results obtained by RNA-Seq, gene expression was assessed by qRT-PCR in three additional pairs of high/low SCR derived embryo pools. Total RNA was extracted from each embryo pool (*n* = 14–54 blastocysts) using an RNaqueous Micro-Kit- (Life Technologies) and cDNA was generated using an iScript cDNA synthesis kit (Bio-Rad Laboratories, CA). Equal amounts of cDNA from each pool of blastocysts were used to generate a pool of cDNA representative of embryos derived from high fertility or low fertility sires. Primers for qRT-PCR reactions were designed to span exon-exon junctions to minimize amplification of genomic DNA, where the sequences are listed in Additional file 1: Table S1. The reference gene, glyceraldehyde 3-phosphate dehydrogenase (*GAPDH)*, was used as in our previous work [17, 23, 24] it was found to be the most stable in blastocyst embryos following a stability test described by Vandensompele et al. [25]. Primers and cDNA were combined with a SYBR green mastermix (iQSYBR Green Supermix kit; Bio-Rad Laboratories, CA) and reactions were carried out using a BioRad iCycler. Expression data was analyzed using the 2^-ΔΔCt^ method by Livak and Schmittgen [26] to calculate the fold difference in expression between samples.

### Supplementation of antisense gapmer of TFB2M to the culture media of presumptive zygotes

As a proof-of-concept, the effect of a differentially expressed gene on embryonic development was assessed using an antisense oligonucleotide, gapmer, supplemented to the culture medium of presumptive zygotes. Gapmer supplementation to media is an effective means to reduce gene expression as cells effectively take up the antisense oligonucleotide and specifically target an mRNA for degradation [27–29]. The gene *TFB2M* was chosen as a target as it was more highly expressed in embryos derived from high fertility sires and expression was validated. The *TFB2M* gapmer sequence (5’-ACGGTAAATGGTCTA-3’) was designed by and purchased from Exiqon, Inc. (Woburn, MA, USA). Embryos were generated by IVF as aforementioned. At the time point in which the presumptive zygotes were placed into culture media, either 1 μM gapmer, 5 μM gapmer, or water (vehicle of gapmer; deemed the control and added at an equal volume as the gapmer) was supplemented to the medium. On day 8 of development, fertilization rate and blastocyst rate were assessed for each of the gapmer supplemented experimental groups as well as the control. Blastocysts were pooled and collected for each experimental group. To assess gene expression following supplementation, total RNA was extracted, cDNA was generated, and qRT-PCR was carried out utilizing the same methodology as described above for gene expression validation. Statistical analysis was performed using the program OriginLab (OriginLab Corporation, Northhampton, MA) in which a paired *t*-test was used to compare the ΔCt values between blastocyst samples for each gene.

### Extraction of DNA from sperm

DNA was extracted using a phenol:choloroform extraction method [30]. Extracted DNA was quantified and quality checked using a Nanodrop ND-1000 spectrophotometer (Nanodrop Technologies, Montchanin, DE). Equal amounts of DNA for each bull were used to generate three respective pools for high and low fertility bulls (*n* = 2 bulls per pool, with the exception of 1 high pool of *n* = 1 bull).

### Affinity purification of methylated DNA regions

To capture differences in methylated regions, a methyl binding domain capture assay combined with next generation sequencing method was employed. A MethylCap kit (Diagenode, Denville, NJ) was used to purify methylated DNA based on high-affinity binding of methyl domain binding proteins. In brief, DNA was dissolved in GenDNA TE to a concentration of 0.1 μg/μl. DNA was then cut into 300–500 base pair fragments using a Bioruptor® sonicator (Diagenode) and was then run on an agarose gel to confirm the presence and size of the fragmented DNA. Fragmented DNA was captured per kit recommendation using magnetic beads to wash unbound DNA followed by elution. Eluted DNA was purified using a MiniElute PCR Purification kit (Qiagen, Germantown, MD). To assess the enrichment of methylated DNA, qRT-PCR was used where duplicates of each sample were tested using the iCycler iQ PCR system (Bio-Rad, Hercules, CA). The relative fold enrichment levels were calculated following the 2^-ΔΔCt^ method; which compares enrichment values of a positive (*TGFB3*) to a negative (*MON2*) primer pair, between experimental and input DNA samples.

### Library preparation and MBD-Sequencing

To prepare the sequencing libraries, fragmented DNA was end repaired using a NEBNext® End Repair Module (NEB, Ipswich, MA) followed by addition of a 3’A to the repaired end of DNA using DNA Polymerase I, Large (Klenow) Fragment (NEB). Paired Solexa adaptors were ligated to the repaired ends of DNA by T4 ligase (Promega, Madison, WI). The DNA was loaded onto an agarose gel, and DNA fragments containing adaptors were selected that were between 200 and 500 bp in size. PCR of the selected DNA fragments was performed using Phusion® Hot Start High-Fidelity DNA Polymerase (NEB) to enrich the purified DNA. The library DNA was quality checked and then quantified using a Qubit Fluorometer (Life Technologies). Cluster generation and sequencing were then performed using a Solexa 1G Genome Analyzer (Illumina Inc., San Diego, CA) following the manufacturer’s recommendations.

### MBD-Sequencing analysis

FASTQ sequence files were examined for quality assurance. After a satisfactory quality confirmation, files were aligned to the bosTau6 (Bos_taurus_UMD_3.1) reference genome obtained from the UCSC browser (http://genome.ucsc.edu). For the alignment process, Bowtie (Ultrafast, memory-efficient short read aligner) was employed [31]. Original fragments consisted in 50 nucleotides although the first 10 5’ and 5 3’ nucleotides of each segment were trimmed for high sequence accuracy. Data manipulation, filtering, and format transformation have been achieved employing a combination of procedures imbedded in SAMtools and BEDtools [32, 33]. Duplicated reads have been removed applying the bRemoveDuplicates option included in the DiffBind package. This action would influence downstream analyses and is critical for the method that we adopted.

The peak-calling step was performed independently in each sample using Model Based Analysis of ChIP-Seq (MACS) [34]. The software empirically models the shift size of the tags and uses a dynamic Poisson distribution to account for local bias, generating more reliable estimates. The differentially methylated regions (DMRs) have been detected with the DiffBind package implemented in R [35, 36] which computes differentially bound sites using affinity data. The input for DiffBind consists of the set of peaks previously identified in MACS and the bam files containing aligned reads for each sample. The program generates a matrix with the consensus peaks; which have been determined from a “minimum overlap” of 3 (the number of replications in the experiment). After setting a contrast between conditions, DiffBind runs an edgeR analysis, which is an empirical Bayes method [24]. For normalization, the method trimmed mean of M-values (TMM) that subtracts the controls reads and considers the effective library size (reads in peaks), was applied. The threshold for DMR calling was set to < 0.1 FDR. In order to annotate the DMRs, the software ChIPpeakAnno has been implemented [37]. ChIPpeakAnno specifies the location, overlaps, relative position and distances for the inquired feature. The annotation information corresponds to bosTau6, the genome used for alignment.

### Validation of differentially methylated regions by bisulfite conversion and sequencing

DNA was extracted as described above from an additional semen straw for each high and low SCR bull. DNA was pooled for high and low fertility bulls, respectively, and each pool was bisulfite converted utilizing an EZ DNA Methylation-Lightning™ kit (Zymo Research, Irvine, CA). As per kit recommendation, a total of 500 ng of DNA per pool were used as input for bisulfite conversion. The bisulfite converted DNA was amplified by PCR for 35 cycles using primers listed in Additional file 2: Table S2. The amplified product was used as a template for a second PCR amplification reaction of 35 cycles. The PCR product was gel purified using an illustra™ GFX™ PCR DNA and Gel Band Purification kit (GE Healthcare Biosciences, Pittsburgh, PA). The purified products were ligated into the pGEM-T Vector (Promega), and transformed into JM109 competent cells (Promega). White bacterial colonies, indicating transformation of the vector, were collected and screened for the DMR of interest by PCR. The PCR products were then Sanger sequenced to analyze the bisulfite-converted sequences. The number of clones analyzed were 31 and 39 from high and low fertility sires, respectively, for CHR19, and similarly 28 high fertility and 30 low fertility derived clones were analyzed for CHR12. The methylation status was determined from each clone and methylation level was summed for all clones for high and low sires to determine the percent of methylated bases at each CpG site. Statistical analysis was performed using Fisher’s Exact Test with the software program R.

## Results

### Development and morphology are similar between embryos generated from high and low fertility sires

To assess whether morphology and development are different between embryos generated from high and low fertility sires, IVF was carried out in two biological replicates for each high and low bull pair for a total of six pairs. Herein, a bull pair will refer to one IVF replicate in which oocytes were randomly split and fertilized with either a high or low SCR sire. In terms of preimplantation embryonic development, embryos that were fertilized with either a high or low SCR sire did not differ in fertilization rate or blastocyst rate (Table 1). The cleavage rate, calculated as the percentage of oocytes that fertilized and cleaved, was comparable (*P* > 0.05) between all SCR sires as 70.28% of the oocytes fertilized with a high SCR bull cleaved and 72.74% of the oocytes cleaved following fertilization with a low SCR bull. Similarly, the blastocyst rate or the percentage of cleaved embryos that developed to the blastocyst stage was not significantly different between high and low SCR bulls as the rates were 29.41% and 27.01%, respectively. Notably, the blastocysts derived from high and low SCR bulls were of similar morphology. Blastocysts of stage 7, grades 1 and 2 as morphologically described by Bo and Mapletoft [19] were collected for further transcriptomic evaluation.

**Table 1:** Development of embryos derived from high and low SCR sires

	Total oocytes	Mean cleavage rate	Mean blastocyst rate
High	2962	70.28% (50.7–84.3)	29.41% (12.7–42.1)
Low	2795	72.74% (61.9–83.6)	27.01% (13.1–36.6)

Embryonic development is represented by the mean rate and the range across 2 IVF replicates per bull, with *n* = 6 high and *n* = 6 low SCR bulls. No significant differences were observed for any development measure between high vs. low fertility sires

### Characterization of the embryonic transcriptome by RNA-Seq

Given that morphology and development rates were similar between embryos produced from high and low fertility bulls, it was intriguing to test whether or not the transcriptomic profiles of these embryos were different. The embryonic transcriptome was profiled at the blastocyst stage through RNA-Seq. A summary of the read alignments is illustrated in Table 2. The percent of uniquely mapped reads from embryos derived from high fertility sires ranged from 47.96 to 63.88% and was comparable to the mapped reads from embryos derived from low fertility sires which ranged from 50.86 to 57.95%. A small portion of reads in embryos derived from both high and low fertility sires mapped to multiple loci or were too short to align (Table 2). The greatest proportion of uniquely mapped reads aligned to exons for embryos derived from high (45.64%) and low (50.10%) fertility sires (Additional file 3: Table S3). Across all samples, the transcripts mapped to a total of 16,710 genes. Differential expression analysis was performed to determine if the embryonic transcriptome differed between those fertilized with sires of varying field fertility. A total of 98 genes (FDR < 1%) were found to be differentially expressed between embryos derived from high and low fertility sires, where 65 genes were more highly expressed in high SCR derived embryos and 33 genes were more highly expressed in low SCR derived embryos. At an FDR < 5%, the number of differentially expressed genes increased to 227, where 135 were more highly expressed in embryos derived from high fertility sires and 92 were more highly expressed in embryos derived from low fertility sires (Additional file 4: Table S4). Table 3 includes a subset of the most significantly differentially expressed genes with an FDR < 1% that are upregulated in embryos derived from either high or low fertility sires. These results suggest that transcriptomic differences in embryos arise between those derived from high or low field fertility sires.

**Table 2:** RNA-Sequencing read alignments for embryos derived from high and low fertility sires

Bull pairs		Number of input reads	% of uniquely mapped reads	% of reads mapped to multiple loci	% of reads unmapped: too short	% of reads unmapped: other
Pair 1	High	36,014,608	63.65	13.98	18.51	3.85
	Low	20,458,553	57.95	14.16	17.42	10.46
Pair 2	High	16,598,719	47.96	15.75	28.05	8.23
	Low	11,904,976	50.86	12.51	29.61	7
Pair 3	High	54,099,488	63.88	10.80	24.32	0.99
	Low	12,870,461	54.15	15.31	21.68	8.84

Sequencing data was generated from three pairs of IVF experiments utilizing a high and low SCR sire for each experiment

**Table 3:** Differentially expressed genes between embryos of high and low fertility sires. A subset of 20 enriched genes for each fertility status ranked by the highest to lowest fold change in expression; all detected at an FDR < 1%

Gene symbol	Gene name	Fold change	*P*-value
Highly expressed in embryos of high fertility sires			
ENSBTAG00000040367		36.63	4.7e^−6^
POLL	Polymerase (DNA directed), lambda	14.86	6.3e^−5^
CYCS	Cytochrome C1 somatic	13.85	2.1e^-.9^
MEPCE	Methylphosphate capping enzyme	8.95	7.9e^−5^
TFB2M	Transcription factor B2, mitochondria	7.71	6.3e^−14^
RPS27	Ribosomal protein S27	7.68	4.7e^−10^
APOM	Apolipoprotein M	7.57	3.2e^−8^
ATP6V0E1	ATPase H+ transporting, lysosomal 9 kDa, V0 subunit e1	6.78	1.3e^−11^
SLC25A14	Solute carrier family 25 (mitochondrial carrier, brain) membrane 14	6.32	8.5e^−7^
NDUFA1	NADH dehydrogenase (ubiquionone) 1 alpha subcomplex, 7, 14.5 kDA	6.31	1.3e^−11^
SFXN4	Sideroflexin 4	5.55	1.3e^−7^
RPS20	Ribosomal protein S20	5.53	2.3e^−7^
RPS11	Ribosomal protein S11	5.39	1.3e^−7^
PSMA1	Proteasome (prosome, macropain) subunit, alpha-type 1	5.25	8.0e^−9^
HCFC1R1	Host cell factor c1 regulator (XPO1 dependent)	5.25	1.8e^−5^
DDT	D-dopa-chrome tautomerase	5.17	4.8e^−9^
EBP	Emopamil binding protein (sterol isomerase)	5.05	9.3e^−10^
GABARAP	GABA(A) receptor-associated protein	4.97	9.9e^−11^
TMSB10	Thymosin beta 10	4.88	6.2e^−8^
ENSBTAG00000006383		4.86	1.2e^−9^
Highly expressed in embryos of low fertility sires			
ENSBTAG00000046713		205.21	1.0e^−6^
TTC37	Tetratricopeptide repeat domain 37	130.66	7.5e^−5^
ENSBTAG00000048042		35.43	5.9^−5^
ALKBH2	alkB, alkylation repair homolog 2	16.90	4.3e^−5^
ENSBTAG00000021503		8.30	1.0e^−5^
PHF14	PHD finger protein 14	7.49	5.3e^−5^
SREK1	Splicing regulatory glutamine/lysine-rich protein 1	6.19	2.4e^−7^
SLC16A7	Solute carrier family 16 (monocarboxylate transporter), member 7	5.73	9.2e^−5^
EEA1	Early endosome antigen 1	5.62	4.4e^−5^
BDP1	B double prime 1, subunit of RNA polymerase III transcription initiation factor IIIb	5.36	4.6e^−5^
ANKRD12	Ankyrin repeat domain 12	4.98	5.7e^−7^
ENSBTAG00000011789		4.93	4.0e^−5^
SMC4	Structural maintenance of chromosome 4	4.86	9.9e^−9^
AKAP9	A kinase (PRKA) anchor protein 9	4.62	3.4e^−5^
HMGN5	High mobility group nucleosome binding domain 5	4.57	2.0e^−5^
ENSBTAG00000032360		4.55	3.6e^−9^
GADD45A	Growth arrest and DNA-damage inducible, alpha	4.51	7.7e^−5^
SURF2	Surfeit 2	4.33	5.1e^−5^
CCDC186	Coiled-coild domain containing 186	4.31	4.2e^−5^
NOL7	Nucleolar protein 7, 27kDA	4.28	5.9e^−7^

### Gene expression validation by quantitative real-time PCR

**Fig. 1:**
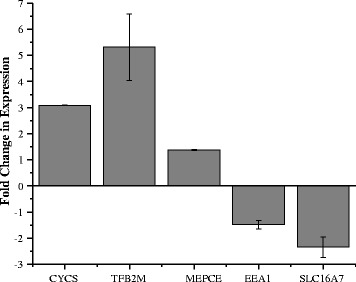
Gene expression validation by qRT-PCR. Expression is represented as the fold change in gene expression in embryos derived from high SCR compared to low SCR sires. Error bars represent the standard error of the mean fold change across 3 qRT-PCR replicates

To confirm the RNA-Seq results, gene expression was tested in three additional pairings of embryos derived from high and low fertility sires. Expression of the genes *CYCS*, *TFB2M*, *MEPCE*, *EEA1*, and *SLC16A7* was assessed by qRT-PCR (Fig. 1). For *CYCS*, *TFB2M*, and *MEPCE*, all of which were more highly expressed in embryos derived from high fertility sires in the RNA-Seq analysis, the fold changes in expression in the biological replicates were 3.09 ± 0.01 SE, 5.32 ± 1.27 SE, and 1.37 ± 0.02 SE, respectively. The genes *EEA1* and *SLC16A7*, which were more highly expressed in embryos of low fertility sires in the RNA-Seq data, were also confirmed by qRT-PCR as the fold changes in expression were higher in low SCR derived embryos, 1.49 ± 0.17 SE and 2.34 ± 0.40 SE, respectively.

### Antisense TFB2M oligonucleotide reduces embryonic development

**Fig. 2:**
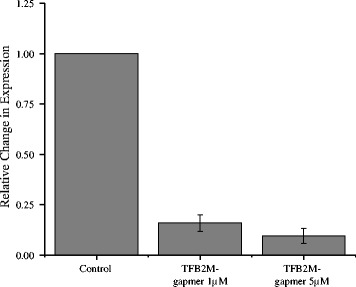
Relative expression of TFB2M in control compared to gapmer supplemented blastocysts. Expression is relative to control blastocysts. Error bars represent the standard error of the mean fold change in expression across *n* = 3 and *n* = 2 IVF replicates for 1 μM and 5 μM gapmer supplemented blastocyst, respectively

To further assess the roles of differentially expressed genes in embryonic development, the *TFB2M* gene was selected as a proof-of-principle for functional analysis because it was a highly expressed gene in embryos derived from high fertility sires and expression was validated by qRT-PCR analysis. The gene was silenced at the zygotic stage using antisense oligonucleotide gapmer technology. The gapmer oligonucleotide is comprised of modified locked nucleic acids (LNA) which flank DNA monomers specific to a target mRNA of interest [27, 28]. Gene silencing is mediated when the gapmer DNA monomers bind to the target mRNA and upon the formation of the DNA:RNA heteroduplex, RNase H will cleave the RNA target strand [27, 28]. Cell culture experiments have demonstrated effective uptake of gapmers from culture media in the absence of transfection agents and efficient repression of gene expression within cells [29, 38]. Following supplementation of 1 μM TFB2M-specific gapmer to the culture media of zygotes, the blastocyst rate of supplemented embryos was significantly reduced by 10.92% (*P* < 0.001), which was about 70% of the control embryos (Table 4). Similarly, blastocyst rate was reduced by 9.58% with 5 μM gapmer (*P* < 0.05) in comparison to control non-supplemented zygotes (Table 4). Further examination of the mRNA expression revealed a significant reduction in gene expression using 1 μM TFB2M gapmer supplemented blastocysts in comparison to controls (*P* < 0.05; Fig. 2). The relative expression of the 5 μM TFB2M gapmer was also greatly reduced and tended towards significance, however, expression was variable across the 2 IVF replicates (*P* = 0.11; Fig. 2).

**Table 4:** Embryonic development following TFB2M gapmer supplementation to presumptive zygotes

Treatment	Total oocytes	Number of unfertilized oocytes	Cleavage rate	Number of blastocysts	Blastocyst rate
Control	330	75	77.27%^a^	93	36.47%^a^
1 μM TFB2M Gapmer	288	61	78.82%^a^	58	25.55%^b^
5 μM TFB2M Gapmer	157	38	75.32%^a^	32	26.89%^b^

Differing superscripts within a column denote statistical significance (*P* < 0.05; Chi-Squared test)

### Evaluation of sperm DNA methylation by MBD-Sequencing

**Fig. 3:**
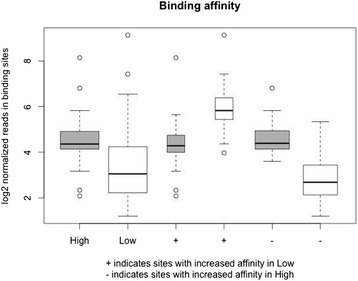
Binding affinity of reads associated with methyl domain proteins between high and low SCR spermatozoa

MBD-Sequencing (MBD-Seq) was performed for three pools derived from high fertility spermatozoa and three pools derived from low fertility spermatozoa, where *n* = 2 bulls per pool and fertility status was based on SCR. Sequencing of DNA regions enriched in methylation sites resulted in a mean of 44,594,169 reads and 52,000,562 reads for high fertility and low fertility pools, respectively. Reads were then aligned to the Bos_taurus_UMD_3.1 reference genome. For all pooled DNA samples, a high percentage of reads aligned to the reference genome, where the total aligned reads was 96.98–97.61% for high pools and 96.27–97.96% for low pools. Figure 3 demonstrates the binding affinity to methyl domain proteins in which significant differences were observed for the overall normalized reads across binding sites between high and low fertility spermatozoa (*P* < 0.0001). Overall, a higher degree of methylation was observed for spermatozoa of high fertility sires as evident by the greater number of methylated binding domains.

### Analysis of differentially methylated regions

**Fig. 4:**
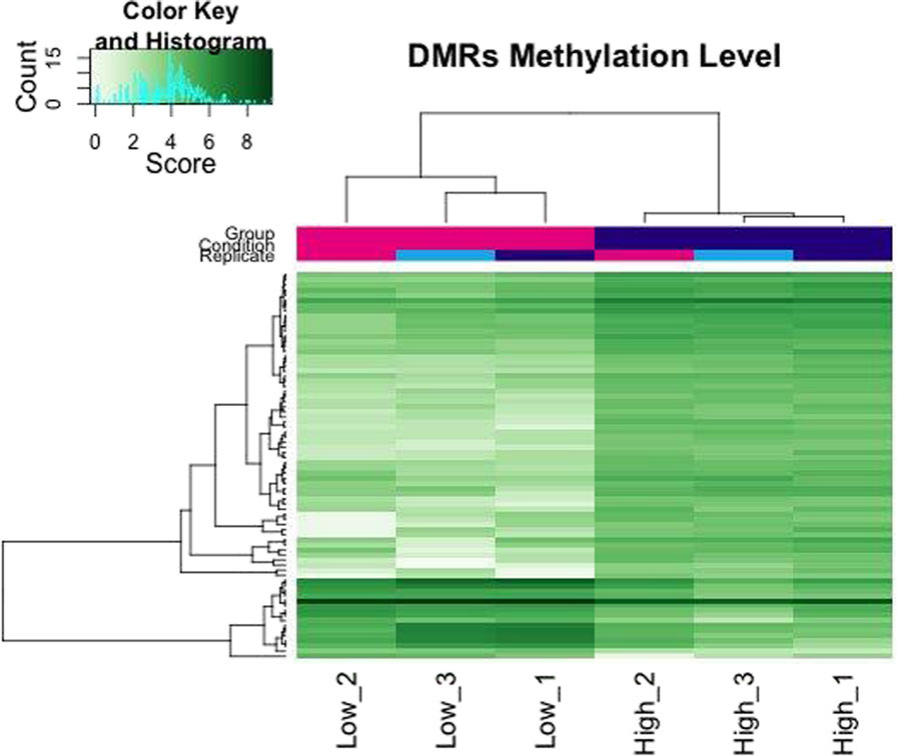
Correlation of DMR methylation levels between pools of high and low SCR spermatozoa. Each row represents an individual DMR and each column represents a pool from either high or low fertility sires. DMRs represented were identified at a FDR of 10%

To determine whether the captured methylated regions differed between high and low fertility sires, analysis of DMRs was performed. The DMRs width ranged from 250 to 3423 bp, with a mean of 521.97 ± 408.52 bp. The DMRs were distributed across 23 chromosomes in which chromosome 5 had the largest number of DMRs (8/76 or 10.5% of the DMRs) and 7 DMRs were unmapped to a specific chromosome (Additional file 5: Figure S1). The DMRs that mapped to unknown regions warrant further investigation and are likely due to poorly annotated regions of the reference genome. Considering the chromosomal length, chromosome 18 had the largest percentage of DMRs (7.5%) followed by chromosome 5 (6.6%), 12 (6.5%) and 15 (4.6%). Differential methylation analysis revealed a total of 76, 40, and 8 DMRs at a 10%, 5% and 1% FDR, respectively (raw data is included in Additional file 6: Table S5). At a 10% FDR, 60 DMRs had enriched methylation levels in sperm of high fertility sires and 16 DMRs had enriched methylation levels in sperm of low fertility sires (Table 5). Figure 4 illustrates the methylation rate at each DMR (FDR 10%), where overall a higher level of methylation was observed across spermatozoa of high fertility sires. Furthermore, principle component analysis confirmed high similarity between pools of similar fertility and dissimilarity between high and low pools as 75% of the variance was explained by fertility status (Additional file 7: Figure S2). Cluster analysis revealed repeatability across samples and a distinct methylation signature between high and low fertility spermatozoa.

**Table 5:** MBD-Seq identification of DMRs located within genes identified at a FDR of 10%

Gene	Chromosome	Region	Gene length	Position in gene DMR		*P*-value
				Start site	End site	
Enriched in high fertility sires						
MMP2	18	23848121–23848499	26,409	19,502	19,880	5.76e^−9^
PLEX2	4	96843179–96843579	546,350	268,810	269,211	1.15e^−8^
LOC100848700	12	71321184–71321564	178,963	42,786	43,167	1.05e^−7^
NXPH1	4	17613149–17613545	374,914	103,807	104,202	1.08e^−6^
EML6	11	37358428–37358736	287,064	69,783	70,092	1.34e^−6^
PIP4K2A	13	24034672–24034997	185,017	133,799	134,124	2.39e^−6^
C5H22orf23	5	110247288–110247609	7926	6034	6356	4.86e^−6^
CTCF	18	35289322–35289651	45,637	44,126	44,456	5.76e^−6^
LOC100848700	12	71373062–71373461	178,963	94,664	95,064	7.71e^−6^
AGBL4	3	9778857–97788873	1,223,677	696,031	696,348	7.98e^−6^
MAGI1	22	35613754–35614143	641,540	117,528	117,918	8.03e^−6^
ST8SIA1	5	88297961–88298379	180,249	11,094	11,513	9.73e^−6^
ANO6	5	35192295–35192666	234,931	135,500	135,872	9.97e^−6^
LOC100848700	12	71363838–71364138	178,963	85,440	85,741	1.14e^−5^
AAK1	11	67807683–67808077	164,724	7241	7636	1.30e^−5^
LOC100848495	24	2838043–2838565	23,590	23,042	23,565	1.44e^−5^
PIP4K2A	13	24018939–24019300	185,017	118,066	118,428	1.34e^−5^
PKHD1	23	24204511–24204877	458,865	408,928	409,295	1.68e^−5^
FGD4	5	77824043–77824292	229,764	220,686	220,936	1.72e^−5^
Enriched in low fertility sires						
ZFYVE28	6	108448662–108452084	98,584	64,266	67,689	2.56e^−9^
KCNK4	29	43213341–43213911	10,180	5101	5672	6.61e^−7^
LOC100296550	15	47960292–47960961	2476			3.03e^−6^
USP40	3	113785622–113786388	89,524	6993	7760	8.86e^−6^
ASPDH	18	57097376–57098221	3087	529	1375	1.16e^−5^
NANOS2	18	53852907–53853452	548	23	569	1.98e^−5^

A total of 25 of the 76 DMRs identified at a 10% FDR were located within a gene and of these 20 DMRs were more highly methylated in spermatozoa of high fertility sires and five DMRs were more highly methylated in spermatozoa of low fertility sires. A greater proportion of the DMRs located within genes were intronic. For example, DMRs in the genes *MMP2*, *CTCF*, *KCNK4,* and *ASPDH* were located within intronic regions, or spanned intronic and exonic portions of the gene body.

### Validation of differentially methylated regions

To validate the MBD-Seq results, methylation was assessed by bisulfite conversion of DNA followed by Sanger sequencing of two DMRs identified by MBD-Seq analysis. DMRs identified on chromosome 12 (CHR12) and chromosome 19 (CHR19) were selected as they represent DMRs that were highly methylated in high fertility bulls and highly methylated in low fertility bulls, respectively. Table 6 reports the level of methylation observed for each CpG site, where 28–39 samples were analyzed per high and low fertility pools for each DMR. The CHR12 DMR had a significantly greater number of methylated CpG sites with 82.1% methylated CpG sites compared to lower fertility sires where 20.6% of the CpG sites were methylated (Table 6, *P* < 0.0001). Comparatively, the CHR19 DMR had a significantly higher level of methylated CpG sites for sperm DNA of lower fertility sires with 34.0% methylated CpG sites, whereas higher fertility sires exhibited 20.8% methylated CpG sites within this DMR (Table 6, *P* < 0.0001). Overall, the MBD-Seq results were validated for both DMRs, thus confirming the observation that sperm DNA of high and low fertility sires differ in their epigenetic signature.

**Table 6:** Validation of MBD-Seq Differential Expression

CpG	DMR on CHR12 (% methylated)		DMR on CHR19 (% methylated)	
	High (*n* = 28)	Low (*n* = 30)	High (*n* = 31)	Low (*n* = 39)
1	80.8	11.1	0	0
2	90.0	24.0	0	0
3	95.5	9.1	17.2	14.7
4	90.0	25.0	16.2	8.1
5	81.8	25.9	6.9	3.1
6	47.8	3.5	11.1	24.3
7	95.0	18.8	26.7	38.9
8	80.8	13.8	38.5	38.5
9	90.0	5.3	9.7	23.9
10	80.8	26.9	9.7	34.3
11	90.0	35.0	10.0	37.8
12	80.8	10.5	21.4	46.2
13	81.5	37.0	22.6	41.0
14	90.0	70.0	26.7	43.6
15			30.0	43.6
16			23.3	65.8
17			34.5	55.6
18			12.9	32.4
19			3.8	28.6
20			22.6	8.1
21			32.3	40.0
22			43.3	51.3
23			37.9	44.4
Total:	82.1%*	20.6%	20.8%	34.0%*

*Denotes a statistically significant difference (*P* < 0.0001) between total methylated CpG sites for a given DMR between high and low fertility spermatozoa. *n* = the number of clones sequenced

The DMRs identified on chromosome 12 and chromosome 19 were assessed for differential levels of methylation by bisulfite conversion followed by Sanger sequencing

## Discussion

Reproductive performance of sires varies greatly in mammals. However, the influence and roles of the paternal genetic component on embryonic development are not well understood. We hypothesized that sires of differing fertility have different epigenetic signatures that affect not only embryonic development, but also the embryonic transcriptome. This study revealed that sire field fertility status did not affect preimplantation embryonic development in terms of both fertilization and blastocyst rate. In contrast, embryos derived from either a high or low fertility sire that were of similar morphology by day 8 of development displayed significant transcriptomic profiles.

Several semen quality parameters such as morphology, motility, and binding ability have been evaluated with limited success for in vitro prediction of sire fertility [39]. Fertility has also been assessed by in vitro development, however, there are discrepancies across studies as to whether field fertility measures are correlated with IVF development [39, 40]. The present study assessed six pairs of high and low fertility bulls where IVF experiments for each pair were conducted twice, and no difference was seen in regards to the fertilization or blastocyst rates between sires of differing SCR. A previous study assessing 21 bulls based on 56-day non-return rate (NRR) found a positive correlation between the field fertility measure of NRR and IVF cleavage and blastocyst rate, however, large variation was also observed across sires [41]. Moreover, studies by Ward et al. [42] and Al Naib et al. [40] also found 150 day NRR and 90 day NRR, respectively, were correlated with cleavage rates. The present study did not observe a correlation between cleavage rate (the percentage of total oocytes which cleaved) and the fertility measure SCR. Lack of differences in cleavage could be explained by initial characterization of each bull’s response to heparin and the ability to capacitate, thereby allowing for optimal fertilization. However, a former study reported no differences between bull fertility and heparin concentration on cleavage rate nor blastocyst rate [43]. Discrepancies in the association between fertility measures and in vitro embryo development reported likely can be attributed to variation in embryo production and analysis methods across different research groups.

Despite similar embryonic development and morphology, RNA-Seq revealed significantly different transcriptomic profiles of embryos derived from differing fertility sires. Many differentially expressed genes were more highly expressed in embryos derived from high fertility sires and functionally have roles in metabolic processes and catalytic activities. For example, the methyl phosphate capping enzyme (*MEPCE*) gene catalyzes the addition of a methyl phosphate cap to 7sk snRNA, a gene that participates in transcription regulation at the transition from initiation to elongation [44, 45]. The transcription factor B2, mitochondrial (*TFB2M*) gene is a mitochondrial transcription factor [46], where overexpression in rat cardiac myocytes resulted in increased mRNA levels of mitochondrial enzymes and increased mitochondrial DNA copy [46, 47]. Another gene related to mitochondrial function is Cytochrome C (*CYCS*), which codes a heme protein that participates in electron transfer within the mitochondrial electron transport chain in addition to promoting apoptosis through activation of Caspase 9 [48–50]. Several of the genes more highly expressed in embryos derived from high fertility sires participate in mitochondrial, and therefore, metabolic function including the aforementioned *TFB2M* and *CYCS*, and also *NDUFA1* [51] and *SFXN4* [52]. Indeed, it has been hypothesized that metabolically “quite” embryos are more viable than those with an ‘active’ metabolism, though the range of values in terms of gene regulation or other markers that determine a level of ‘quietness’ is unknown [53, 54]. Here, the roles of the highly expressed genes identified are not well defined in embryonic development. Thus, these genes are considered new candidates that may influence the embryo’s developmental potential.

Several genes more highly expressed in embryos from low fertility sires may explain poorer development beyond the blastocyst stage. The expression of solute carrier family 16 (monocarboxylate transporter), member 7 (*SLC16A7*), was previously detected in mouse preimplantation development where it acts to shuttle lactate and also plays a role in regulating redox in the early mouse embryo [55]. Moreover, upon glucose deprivation within early mouse embryos, the levels of *SLC16A7* become upregulated during oxidative stress [56]. Upregulation the growth arrest and DNA-damage-inducible, alpha (*GADD45A*) gene also indicates stress as genes within the GADD45 family are stress sensors with roles in DNA repair, cell cycle regulation, and apoptosis as well as DNA demethylation [57, 58]. Therefore, it is plausible that the transcriptome differences observed in embryos derived from low fertility sires may be indicative of poorer developmental outcome.

A limitation of the present study is the number of bull pairings for which embryos were sequenced and analyzed by RNA-Seq. The disparity in the total number of reads obtained was observed between embryos compared within a high/low bull pairing. Moreover, the percentage of uniquely mapped reads is relatively small, which could be an artifact of loss of mRNA during the process of RNA amplification or technical error. Therefore to assess the validity of the differentially expressed genes identified by RNA-Seq, expression was further evaluated by qRT-PCR in three additional bull pairings of embryos. The qRT-PCR results confirmed the RNA-Seq expression results using additional biological replicates.

Another limitation of the study is that the development results are restricted to the preimplantation stage of development, as embryo transfer was not feasible. Variations in embryo implantation rate, miscarriage rate, and the live birth rate could not be evaluated. Interestingly, at the time point in which embryo transfer could take place, embryos derived from different sires presented with similar morphology. Likewise, Driver et al. [59] reported that preimplantation embryonic transcriptome of morphologically similar in vivo and in vitro derived embryos to be strikingly different. While it is well established that in vivo embryos have better pregnancy outcomes compared to their in vitro derived counterpart [60], differences in gene expression profiles could plausibly underlie the embryo’s potential to progress to establishing and maintaining a pregnancy. A study by El-Sayed et al. [61] identified certain gene profiles within embryonic blastocyst biopsies were correlated with pregnancy outcome. Similarly, Ghanem et al. [62] also found in vivo derived blastocyst biopsies to be associated with pregnancy outcome, where both studies identified PLAC8 to be upregulated in transferred blastocysts that resulted in the delivery of a calf. Genes identified in these studies were not found to be differentially expressed in the present study, though here RNA-Seq was utilized whereas the previous studies utilized a microarray strategy. Hence, future work is needed in which embryos derived from sires of differing fertility statuses are biopsied, and the developmental outcome is followed long term to determine developmental potential.

As a proof-of-concept that the differentially expressed genes identified are important to embryonic development, expression knockdown of *TFB2M* was performed by antisense gapmer technology. The gene *TFB2M* was selected for expression knockdown as it may play a role in regulating embryo metabolism through its role in transcription of mtDNA [46]. As the embryo’s genome is activated glycolysis becomes the main mechanism of providing ATP, and oxidative phosphorylation is inhibited to maintain a more quiescent state, which confers a more viable embryo [63]. Gapmer mediated knockdown of *TFB2M* resulted in decreased development to the blastocyst stage. The *TFB2M* gene was more highly expressed in embryos derived from higher fertility sires and gene knockdown demonstrated that reduction in expression leads to reduced embryonic development. Thus, embryos derived from low fertility sires with lower expression could plausibly be developmentally compromised. Interestingly, bovine spermatozoa contain a microRNA, miR-2284x [64], which targets the *TFB2M* mRNA as predicted by the online tool, TargetScan (http://www.targetscan.org/). Further characterization of sperm-derived factors, such as microRNAs, should, therefore, be explored as potential contributors to embryonic reprogramming and may be developed as biomarkers of reproductive performance.

While the transcriptomic landscape depicts embryonic differences influenced by the sire, it is still unclear why or how sire field fertility is correlated with the embryonic transcriptome. Multiple components may attribute to the differences observed within the embryonic transcriptome, including the impact of paternal allelic variation as well as the delivery of paternal factors at the time of fertilization. Several studies have indicated that the “RNA package” delivered at the time of fertilization is strikingly different between bulls of differing fertility [10, 11, 13–15]. However, it is unknown whether the population of RNA delivered to the oocyte or other factors such as epigenetic marks or degree of DNA integrity of differing field fertility sires may contribute to the differences observed in the embryonic transcriptome.

The investigation into the epigenetic signature of the sperm between the high and low fertility sires, revealed 76 regions to be differentially methylated between sires of differing fertility status. Similarly, a study by Verma et al. [65] reported methylation analysis by microarray of high and subfertile buffalo spermatozoa in which 73 genes in high fertility and 78 genes in subfertile spermatozoa were hypermethylated, where pathway analysis characterized these genes to have roles in transcriptional regulation and cell proliferation. Indeed, 13 differentially methylated genes were reported to have functional roles in sperm processing including spermatogenesis and capacitation as well as embryonic development. A study by Camprubi et al. [66] comparing the DNA methylation of spermatozoa from high fertility and infertile human semen donors identified 696 differentially methylated CpG nucleotides associated with 501 genes, where 13 CpG sites were associated with genes plausibly involved in spermatogenesis. Comparatively, there is no overlap between the genes identified in the present study and those in the study by Verma et al. [65] and Camprubi et al. [66]. Altogether, it can be concluded that the DNA methylation levels are strikingly different between spermatozoa of males of differing fertility status and that epigenetic regulation may impact key genes related to sperm processing and embryonic development.

Several DMRs identified between high and low fertility sires are located within genes with functional roles in spermatogenesis and fertilization that may underpin the differences in field fertility. For instance, a study by Ferrer et al. [67] found that MMP2 co-localizes with acrosin on the inner acrosomal membrane of bull spermatozoa, where the authors suggest the protease may mediate sperm penetration at the zona pellucida as matrix metalloproteases function to cleave extracellular matrix components. Another DMR was identified in the *KCK4* gene, a member a two-pore domain potassium (K_2P_) channel family [68], where potassium channels have important physiological roles in the acrosome reaction and fertilization [69]. In bull spermatozoa, a study by Hur et al. [68] reported that the protein of *KCK4* localizes to the equatorial region of acrosome reacted sperm, and that inhibitor of the K_2P_ channels reduces not only fertilization but also development of bovine and mouse embryos in vitro. Interestingly, a DMR was located in the *CTCF* gene, which has been associated with spermatogenesis and male fertility [70, 71]. The *CTCF* gene plays a critical role in genome-wide gene regulation and has roles in epigenetic reprogramming, gametogenesis and embryo development and is also associated with fertility, as reviewed by Franco et al. [70]. Indeed, a study by Hernandez-Hernandez et al. [71] reported that mice with a conditional knock-out of the *CTCF* gene had smaller testis and spermatogenesis was impacted as several males were infertile. Moreover, spermatozoa of the CTCF-conditional knock-out mice also demonstrated aberrant histone retention and disrupted chromatin compaction. While the functions of the DMRs identified within the present study remain to be elucidated, several of the DMRs identified are within genes associated with roles in spermatogenesis, fertility, and embryonic development.

It is well understood that during the process of spermatogenesis the chromatin structure undergoes drastic remodeling by replacing histones with protamines to achieve a high condensation of DNA. Also, sites, where histones are retained, are within key developmental gene regions [72]. Errors within spermatogenesis relating to the condensation of the DNA as well as maintenance of epigenetic marks could plausibly explain the differences in embryonic gene expression. Indeed, less DNA condensation, protamine exchange, and higher DNA damage have been observed in spermatozoa of lower fertility bulls in comparison to higher fertility bulls [73, 74]. Therefore, future studies should focus on better identification of spermatogenesis errors as well as on epigenetic marks that have an effect on the embryonic transcriptome and if these errors are associated with fertility status and developmental outcome.

## Conclusions

Male fertility had received less attention in comparison to female fertility, yet it has been demonstrated that the male gamete contributes not only DNA but also RNA and signaling factors to the oocyte at fertilization. The present study identified transcriptomic differences within embryos derived from bulls of differing fertility status at the preimplantation stage of development. While transcriptomic differences within the embryos were observed at the preimplantation stage, the association between male fertility and embryonic development following embryo transfer should be investigated in the future. In addition, it is vital to explore whether DNA condensation and integrity as well as alterations in epigenetic signatures within the spermatozoa contribute to sire fertility and its effect on embryo development.

## Additional files

Additional file 1: Table S1. Primer sequences utilized for qRT-PCR gene expression analysis to validate the RNA-Seq results. (DOC 33 kb)

Additional file 2: Table S2. Primer sequences for PCR of bisulfite-converted DNA. (DOC 29 kb)

Additional file 3: Table S3. Genomic distribution of aligned reads in embryos derived from high and low fertility bulls. Represented as a percentage of the total mapped reads. (DOC 27 kb)

Additional file 4: Table S4. Differentially expressed genes between embryos derived from high and low fertility sires (FDR <5%). (DOCX 27 kb)

Additional file 5: Figure S1. Distribution of the DMRs across chromosomes. The histogram represents the number of DMRs located on each chromosome. unk: represents regions with an unknown location as they do not map to a chromosome. (PDF 34 kb)

Additional file 6: Table S5. Differentially Methylated Regions between high and low SCR sires (FDR <10%). (XLS 40 kb)

Additional file 7: Figure S2. Principal component analysis for high and low fertility pools. (PDF 21 kb)

## Abbreviations

COC: Cumulus oocyte complex
DMR: Differentially methylated region
FAF-BSA: Fatty acid-free bovine serum albumin
FDR: False discovery rate
IVF: In vitro fertilization
LNA: Locked nucleic acid
MBD-Seq: MBD-sequencing
miRNA: microRNA
NRR: Non-return rate
qRT-PCR: Quantitative real time PCR
RNA-Seq: RNA-sequencing
SCR: Sire conception rate
TALP: Tyrode's albumin lactate pyruvate
TMM: Trimmed mean of M-values
TPM: Transcript per million
