# Supplementary material for: Male fertility status is associated with DNA methylation signatures in sperm and transcriptomic profiles of bovine preimplantation embryos
Source: BMC Genomics. 2017 Apr 5;18:280. doi: 10.1186/s12864-017-3673-y (PMC5382486; doi:10.1186/s12864-017-3673-y)
Supplement: Supplementary file 1 — Primer sequences utilized for qRT-PCR gene expression analysis to validate the RNA-Seq results. (DOC 33 kb) [file 12864_2017_3673_MOESM1_ESM.doc]

**Table S1.** Primer sequences utilized for qRT-PCR gene expression analysis to validate the RNA-Seq results

| **Gene** | **Primer Sequence**  **(5’–3’)** | **Amplicon Size**  **(bp)** |
| --- | --- | --- |
| *CYCS* | F: GCGCACAGAGAAAGAACGATTT | 168 |
|  | R: AGAATCCAGGAGCCTGACCT |  |
| *EEA1* | F: GCGTTGACTAGAAAGTGGGC | 91 |
|  | R: AGTGATGCCGTCTCACTGTT |  |
| *SLC16A7* | F: GAGGACTCGTCCAGGGACATA | 120 |
|  | R: GATTAACTGCTGGTCTCCGGC |  |
| *TFMB2* | F: GGAGTGAAAGCACATCCTTGG | 186 |
|  | R: CGTTAGTTTCCGGCATTCCT |  |
| *MEPCE* | F: CGCCAGAACATCCGACACTA | 239 |
|  | R: TCCAGCACATAGTTACCCGTG |  |
| *GAPDH* | F: TGCCCAGAATATCATCCC | 134 |
|  | R: AGGTCAGATCCACAACAG |  |
