# Supplementary material for: Male fertility status is associated with DNA methylation signatures in sperm and transcriptomic profiles of bovine preimplantation embryos
Source: BMC Genomics. 2017 Apr 5;18:280. doi: 10.1186/s12864-017-3673-y (PMC5382486; doi:10.1186/s12864-017-3673-y)
Supplement: Supplementary file 2 — Primer sequences for PCR of bisulfite-converted DNA. (DOC 29 kb) [file 12864_2017_3673_MOESM2_ESM.doc]

**Table S2.** Primer Sequences for PCR of Bisulfite-Converted DNA

| **DMR ID**  **(chromosome: position)** | **Primer Sequence**  **(5’–3’)** | **Amplicon Size (bp)** |
| --- | --- | --- |
| *CHR 19:*  53339907-53340346 | F: GAGGTTTAGGAGTGGAGTTTTTAATTA | 348 |
|  | R: AAAAAACAAAACATCCCAAAATAAC |  |
| *CHR 12:*  71321184-71321564 | F: TGGGAGGTGAGATTTGGTATATAAA | 325 |
|  | R: CAACAAAAACCAACAATATCAAAC |  |
