# Supplementary material for: Male fertility status is associated with DNA methylation signatures in sperm and transcriptomic profiles of bovine preimplantation embryos
Source: BMC Genomics. 2017 Apr 5;18:280. doi: 10.1186/s12864-017-3673-y (PMC5382486; doi:10.1186/s12864-017-3673-y)
Supplement: Supplementary file 3 — Genomic distribution of aligned reads in embryos derived from high and low fertility bulls. Represented as a percentage of the total mapped reads. (DOC 27 kb) [file 12864_2017_3673_MOESM3_ESM.doc]

**Table S3.** Genomic distribution of aligned reads in embryos derived from high and low fertility bulls. Represented as a percentage of the total mapped reads

| **Bull** | **3’-UTR Exons** | **5’-UTR Exons** | **Coding Exons** | **Sum of Exons** | **Intron** | **rRNA** |
| --- | --- | --- | --- | --- | --- | --- |
| High | 17.09 | 0.80 | 27.75 | 45.64 | 13.65 | 0.01 |
| Low | 13.67 | 1.49 | 34.94 | 50.10 | 11.36 | 0.01 |
