# Supplementary material for: Male fertility status is associated with DNA methylation signatures in sperm and transcriptomic profiles of bovine preimplantation embryos
Source: BMC Genomics. 2017 Apr 5;18:280. doi: 10.1186/s12864-017-3673-y (PMC5382486; doi:10.1186/s12864-017-3673-y)
Supplement: Supplementary file 4 — Differentially expressed genes between embryos derived from high and low fertility sires (FDR <5%). (DOCX 27 kb) [file 12864_2017_3673_MOESM4_ESM.docx]

| "ID" "name" "logFC" "logCPM" "LR" "PValue" "FDR" |
| --- |
| "48" "ENSBTAG00000000072" "TFB2M" 2.94673292254127 9.5722056231748 56.2610302619706 6.34608204738031e-14 6.26485219717385e-10 |
| "9957" "ENSBTAG00000015100" "ATP6V0E1" 2.92062756142764 11.2317805423085 45.9681287679939 1.20192522171665e-11 4.21067448813453e-08 |
| "22581" "ENSBTAG00000046319" "NDUFA7" 2.65729304703674 9.09181450832633 45.845478971085 1.27958098302305e-11 4.21067448813453e-08 |
| "9806" "ENSBTAG00000014883" "GABARAP" 2.3141411998869 10.8501624972051 41.8472292401182 9.86905241268916e-11 2.43568213545169e-07 |
| "9162" "ENSBTAG00000013924" "RPS11" 2.42902008103055 12.9910538481131 41.3733092585388 1.25762864838161e-10 2.48306200336464e-07 |
| "5762" "ENSBTAG00000008713" "WDR21A" 2.65992113139057 7.90905296048301 40.6674559848959 1.80467865672424e-10 2.96929794986362e-07 |
| "10296" "ENSBTAG00000015598" "RPS10" 2.20637562605881 11.6863765476867 38.9244210970136 4.40534911272785e-10 5.80824394382679e-07 |
| "9125" "ENSBTAG00000013866" "RPS27" 2.94030410817329 14.2854495613773 38.7951982556415 4.70684274216109e-10 5.80824394382679e-07 |
| "6134" "ENSBTAG00000009287" "EBP" 2.33675764264124 8.91415457286681 37.4623533129956 9.31945540775206e-10 1.02224070872587e-06 |
| "4217" "ENSBTAG00000006383" "nan" 2.18989583841384 11.043916597927 36.9265594162142 1.22663570002784e-09 1.21093476306748e-06 |
| "14791" "ENSBTAG00000023823" "CYCS" 3.79130112730559 8.13758692414356 35.8704798555411 2.10879699383567e-09 1.8925494475587e-06 |
| "16688" "ENSBTAG00000032360" "nan" -2.18629748142877 9.29644631876258 34.8523184609158 3.55685754819444e-09 2.92610814298129e-06 |
| "19018" "ENSBTAG00000040367" "nan" 5.19509577858067 5.14703135864452 34.3167043775282 4.68344593610793e-09 3.39527490696974e-06 |
| "14471" "ENSBTAG00000022027" "DDT" 2.36901940463664 8.79968150973151 34.2627853223075 4.81501708849032e-09 3.39527490696974e-06 |
| "4340" "ENSBTAG00000006564" "PSMA1" 2.39357547439928 11.2844788050745 33.2691945718221 8.0243774772208e-09 5.28111029700825e-06 |
| "3882" "ENSBTAG00000005862" "SMC4" -2.28193617229512 8.02848486995532 32.8585821584316 9.91125502335189e-09 6.11524434940812e-06 |
| "5128" "ENSBTAG00000007754" "NDUFA3" 2.23862114389878 8.79674662205456 31.6555634981275 1.84086471136729e-08 1.06900096650694e-05 |
| "5837" "ENSBTAG00000008833" "APOM" 2.9309013952237 5.62212536514546 30.5907949092748 3.1860175540852e-08 1.74735362744051e-05 |
| "3743" "ENSBTAG00000005654" "TMSB10" 2.28579914231343 12.114028649064 29.3078933821703 6.1743669513402e-08 3.20807108124371e-05 |
| "5774" "ENSBTAG00000008730" "SFXN4" 2.47330062365433 7.35237606331158 27.8905729061807 1.28373701072878e-07 6.33652588495726e-05 |
| "12566" "ENSBTAG00000019147" "RPS20" 2.46614623872675 14.768742016639 26.7327134158541 2.3363246749676e-07 0.000106801010508188 |
| "4839" "ENSBTAG00000007321" "SREK1" -2.62897441169529 6.2855793936984 26.6968538604724 2.38008734925054e-07 0.000106801010508188 |
| "23799" "ENSBTAG00000047537" "CCAR1" -1.97524688839192 7.63465072730108 25.8865273885526 3.62087510023568e-07 0.000155414256476203 |
| "22233" "ENSBTAG00000045971" "nan" 1.9688228976229 7.73553552943884 25.5164868366808 4.38618794924527e-07 0.000180418530978955 |
| "1857" "ENSBTAG00000002755" "ANKRD12" -2.31712967066949 6.77130706581203 25.0036484050043 5.72219341177221e-07 0.000223724695024357 |
| "14157" "ENSBTAG00000021554" "NOL7" -2.09762429980064 8.21198051067633 24.9471807489318 5.89226303751346e-07 0.000223724695024357 |
| "8003" "ENSBTAG00000012117" "ATP6AP1" 1.79366412684958 10.5015350133237 24.7958167140494 6.37356422696801e-07 0.000233036392772697 |
| "22382" "ENSBTAG00000046120" "nan" 1.96142919588211 7.60317863046527 24.3307324733428 8.11342227876353e-07 0.000286056088342691 |
| "10723" "ENSBTAG00000016263" "SLC25A14" 2.76076915611696 5.97632199698397 24.2448466650792 8.4833758895159e-07 0.000288785816487245 |
| "22975" "ENSBTAG00000046713" "nan" -7.68093893373116 3.24963901905469 23.8698272828322 1.03074726445317e-06 0.00033918456648939 |
| "6168" "ENSBTAG00000009345" "AMZ2" 1.97235692264542 7.66573315231927 23.6757826870733 1.14008194765266e-06 0.000363060935071841 |
| "15517" "ENSBTAG00000027361" "ROMO1" 2.06344710311282 7.8162544372746 23.4767760437459 1.26430760428897e-06 0.000390038895923148 |
| "23476" "ENSBTAG00000047214" "TRAPPC1" 2.13433179990054 6.36372592399706 23.1605552728735 1.49022946486057e-06 0.000445804402336471 |
| "1386" "ENSBTAG00000002060" "RPL19" 2.12746692072093 11.8128304627642 22.8725666433591 1.73104934428633e-06 0.000502615268435136 |
| "3820" "ENSBTAG00000005765" "SYNGR1" 2.15715246479886 7.49365748180997 22.7694515393512 1.82646032520423e-06 0.000515166180869033 |
| "3598" "ENSBTAG00000005414" "nan" 2.2806901951119 8.66339167314153 22.5662921987092 2.03015511248859e-06 0.000548520940685376 |
| "14304" "ENSBTAG00000021785" "TMEM219" 1.75998149203596 8.77827028146977 22.5178650416868 2.08198299854803e-06 0.000548520940685376 |
| "9045" "ENSBTAG00000013723" "MRPS25" 2.24256357922484 7.41180473143199 22.4909083882254 2.11140556584727e-06 0.000548520940685376 |
| "6539" "ENSBTAG00000009904" "SPIC" -1.93766276495918 7.05554187506818 22.0639600960885 2.63714756363111e-06 0.000667536429440163 |
| "18642" "ENSBTAG00000039555" "COX7C" 1.70380654622236 10.8187540834046 21.4586168607957 3.61547632241074e-06 0.000892299556370971 |
| "12184" "ENSBTAG00000018542" "COX5B" 2.1551079118375 11.0604643336721 21.3825168839081 3.7618594192575e-06 0.000905782346022197 |
| "8882" "ENSBTAG00000013461" "RPL24" 1.8556167170724 13.4732418933291 20.8132362545423 5.06318275526081e-06 0.00119008905142702 |
| "2150" "ENSBTAG00000003205" "RPL35" 1.78828251238777 12.4013068719171 20.5717459968595 5.74375944223426e-06 0.0013186603072962 |
| "5621" "ENSBTAG00000008493" "AQP3" 1.68421135563758 10.1929378075135 20.1057062979812 7.32777198490494e-06 0.001635517611684 |
| "14697" "ENSBTAG00000023343" "RPL28" 1.87077356377649 10.1313953777652 20.06073592476 7.50211877140947e-06 0.001635517611684 |
| "16554" "ENSBTAG00000031875" "BANF1" 1.54445025385112 8.99832329536853 20.0306875746439 7.62092890371394e-06 0.001635517611684 |
| "5562" "ENSBTAG00000008403" "ROCK1" -1.74998967323662 7.70647364603542 19.4995189457924 1.00625020437103e-05 0.00208525568323439 |
| "14126" "ENSBTAG00000021503" "nan" -3.05261638770275 7.55923557574601 19.4850511201007 1.01390065635383e-05 0.00208525568323439 |
| "7439" "ENSBTAG00000011299" "nan" 2.12668381417761 6.35164228250045 19.3897470221605 1.06577715175731e-05 0.00214721470247922 |
| "14727" "ENSBTAG00000023471" "RPL36" 1.964996418995 7.66121557104227 19.2954744006525 1.1197143321265e-05 0.00221076397735055 |
| "4496" "ENSBTAG00000006777" "TLCD1" 1.93211390021966 7.49314942021333 19.0394133920203 1.28046167503476e-05 0.00247857208940061 |
| "13089" "ENSBTAG00000019913" "DNPH1" 1.95731065835776 7.53294947723122 18.7616555467608 1.48115330361779e-05 0.00281191257948362 |
| "7761" "ENSBTAG00000011787" "ISCU" 1.48067941454213 8.78443035212278 18.5312980375946 1.67137099445178e-05 0.00308424377086318 |
| "1021" "ENSBTAG00000001520" "MRPL24" 1.90871710396618 7.23473635334654 18.5033415048195 1.6960668093536e-05 0.00308424377086318 |
| "10964" "ENSBTAG00000016637" "WBP4" -2.05038911136016 6.91665360111011 18.4784885021639 1.71832868109274e-05 0.00308424377086318 |
| "18590" "ENSBTAG00000039442" "SEC62" -1.88343702311876 7.07551790844953 18.4060134755308 1.78493786531218e-05 0.00314659046542177 |
| "1594" "ENSBTAG00000002367" "HCFC1R1" 2.39121753833179 5.18510869281713 18.3411736043143 1.84672545758577e-05 0.0031983988977696 |
| "13003" "ENSBTAG00000019794" "SYPL1" 1.50183841784972 9.33131696152913 18.2632581594408 1.92382233917276e-05 0.00327447829867474 |
| "1263" "ENSBTAG00000001877" "HMGN5" -2.19189283952242 6.4374971652045 18.2144169388901 1.97379089721008e-05 0.00330066849979352 |
| "10440" "ENSBTAG00000015831" "RPL18A" 1.72453019342813 11.9431432034777 18.1835136384311 2.00607890992313e-05 0.00330066849979352 |
| "13715" "ENSBTAG00000020852" "SLC16A11" 1.91371408708905 6.4057539430823 18.1455359269816 2.04648601145271e-05 0.00331195244345264 |
| "1338" "ENSBTAG00000002000" "TIMM8B" 1.5150976751949 8.58509359081989 18.0779105872291 2.12047244057251e-05 0.00337633934408577 |
| "7412" "ENSBTAG00000011252" "RAD50" -1.87342296535804 8.58096717035797 17.7174216965822 2.56268780487803e-05 0.00401569111265967 |
| "1202" "ENSBTAG00000001794" "RPL36" 1.70081033325483 9.40698158669919 17.3620253088653 3.08937296477913e-05 0.0047653577981718 |
| "6460" "ENSBTAG00000009783" "nan" 1.77347179112239 7.63743326498153 17.2764404092473 3.23168988893309e-05 0.00490819116669962 |
| "4927" "ENSBTAG00000007442" "AKAP9" -2.20930627416026 6.84943103629949 17.1886312415285 3.38455803683444e-05 0.0050624783241863 |
| "2877" "ENSBTAG00000004316" "BOD1L" -1.77047873155177 6.69694561104818 17.1227724398056 3.50396466462862e-05 0.00510141394902865 |
| "20560" "ENSBTAG00000043949" "PAWR" -2.09113061259795 6.4006721683349 17.1173730707604 3.51393991626771e-05 0.00510141394902865 |
| "4065" "ENSBTAG00000006134" "DYNLRB1" 1.91426680899765 7.15753224447064 17.0725877102583 3.59778821190595e-05 0.00511273131786467 |
| "1122" "ENSBTAG00000001671" "RTF1" 1.93037726846942 9.64300248835487 17.0581129624745 3.62531596688135e-05 0.00511273131786467 |
| "5010" "ENSBTAG00000007572" "ATP6V1F" 1.40254127506083 8.9724004067506 16.9558097446793 3.82600547439343e-05 0.0053197642314383 |
| "7763" "ENSBTAG00000011789" "MGC157163" -2.30144311489963 6.55223631651304 16.875380058028 3.99159813112264e-05 0.00546990341803994 |
| "6543" "ENSBTAG00000009908" "RPS3A" 1.48465515306518 14.2313448072519 16.8502486680316 4.04480297322646e-05 0.00546990341803994 |
| "13296" "ENSBTAG00000020233" "CCDC186" -2.10813096052982 6.89252379526807 16.7600960490666 4.24159688121269e-05 0.0055416194920727 |
| "10600" "ENSBTAG00000016079" "COX4I1" 1.53028973354934 9.82123461635389 16.7571879227197 4.24810283687281e-05 0.0055416194920727 |
| "13041" "ENSBTAG00000019846" "ALKBH2" -4.0789452359553 5.23616052882822 16.7491048814512 4.26623866893766e-05 0.0055416194920727 |
| "285" "ENSBTAG00000000421" "EEA1" -2.48962517051755 5.8965548049684 16.7003223294296 4.37735604841611e-05 0.00561211154674855 |
| "17328" "ENSBTAG00000035370" "TBPL1" 2.0978571186635 6.58558669185343 16.6684849732441 4.45144009269328e-05 0.00563392520449591 |
| "245" "ENSBTAG00000000363" "BDP1" -2.42318634346952 5.8389613459992 16.608719661685 4.59393276209985e-05 0.00574067142119616 |
| "10762" "ENSBTAG00000016315" "COTL1" 1.3343619287394 10.1556465290865 16.5512935822846 4.73516735132824e-05 0.00584319651153905 |
| "14239" "ENSBTAG00000021688" "FH" 1.46105131228099 8.32935156062246 16.5255315915768 4.7999375619715e-05 0.00584999797676329 |
| "30" "ENSBTAG00000000046" "SURF2" -2.11383775948852 6.54930658818348 16.4284377552578 5.05215748662806e-05 0.00608230472048686 |
| "3257" "ENSBTAG00000004913" "RNF167" 1.96008912441925 6.40579892930382 16.3832665813603 5.17399947310513e-05 0.00615394250584263 |
| "7785" "ENSBTAG00000011819" "PHF14" -2.90535204381551 4.8741341462856 16.3369633026795 5.30196288370728e-05 0.00623106876047122 |
| "24304" "ENSBTAG00000048042" "nan" -5.14680496683109 3.12948435711577 16.130440136122 5.91260312560947e-05 0.00686696683011961 |
| "2407" "ENSBTAG00000003576" "POLL" 3.89355008677674 2.94328882067733 16.0084500973435 6.30603976826909e-05 0.00723874704562238 |
| "5072" "ENSBTAG00000007662" "GRP78" -1.50922137475171 7.90722154541485 15.9073437645137 6.6520109234974e-05 0.00754812090077773 |
| "396" "ENSBTAG00000000585" "LY6G6C" 1.95372799457463 6.30218499136246 15.718179513126 7.35142116773469e-05 0.00824695792816782 |
| "6023" "ENSBTAG00000009131" "TTC37" -7.02968577096585 2.54575213503618 15.6742815059126 7.52403435568455e-05 0.00828741528283105 |
| "5733" "ENSBTAG00000008664" "EIF2B2" 1.51103646093287 8.0509246863825 15.6664180016931 7.55538265249994e-05 0.00828741528283105 |
| "9122" "ENSBTAG00000013860" "GADD45A" -2.17155598715816 7.22861893249093 15.6410735440677 7.65731676286846e-05 0.00830692649264148 |
| "20257" "ENSBTAG00000043482" "7SK" 3.16150990457302 8.29137238461942 15.5808519318192 7.90510964305665e-05 0.00848252634741905 |
| "22620" "ENSBTAG00000046358" "PABPC1" -1.3886347689251 10.0278217131411 15.5140965532188 8.18922733218868e-05 0.00869290884122223 |
| "242" "ENSBTAG00000000359" "EIF3J" -1.79382578880734 6.8757258545677 15.3889139276663 8.75001434899449e-05 0.00918937677162485 |
| "10916" "ENSBTAG00000016563" "GOLGA4" -1.94312531536203 9.02549465276662 15.3179237090798 9.08504115235825e-05 0.00944079223748217 |
| "13993" "ENSBTAG00000021287" "SLC16A7" -2.51750395642245 5.16871453150506 15.2864898149372 9.23748305193683e-05 0.00949921173840837 |
| "1227" "ENSBTAG00000001827" "GID8" 1.88565685627159 7.74798335773883 15.1959938429197 9.69087929176357e-05 0.00976451526097625 |
| "1221" "ENSBTAG00000001821" "PRRC2C" -1.28984951913941 9.2154196717694 15.1955223617746 9.69329918532893e-05 0.00976451526097625 |
| "18351" "ENSBTAG00000038931" "nan" 1.79546494629146 6.27397122964262 15.0860881048858 0.000102717505257656 0.0102223912521232 |
| "10168" "ENSBTAG00000015388" "RPL18" 1.57151160102518 10.6865574700562 15.0708631490602 0.000103549344126045 0.0102223912521232 |
| "4140" "ENSBTAG00000006247" "DHPS" 1.78248136306292 6.16153360519275 15.0139427936306 0.000106719784262028 0.0104310664379677 |
| "3225" "ENSBTAG00000004872" "MRPL20" 1.39594108365832 7.65780400604548 14.9389436450159 0.000111046894078835 0.0107111455862668 |
| "12962" "ENSBTAG00000019730" "SFRS18" -2.5869552062257 8.37654028524334 14.9269466718757 0.000111755266955579 0.0107111455862668 |
| "60" "ENSBTAG00000000087" "HSD17B12" -1.43335063314116 7.56361642026028 14.8933619151117 0.000113762594265467 0.0107986954864297 |
| "9992" "ENSBTAG00000015145" "S100A11" 1.74563628774238 9.03798303075814 14.838749015359 0.00011710454459735 0.0109080902424818 |
| "5119" "ENSBTAG00000007737" "UBA52" 1.29603246041433 10.1561121208997 14.838420302348 0.000117124956007199 0.0109080902424818 |
| "22793" "ENSBTAG00000046531" "nan" 1.94303432778285 6.59484444798733 14.7403333522893 0.000123378468073941 0.0113831050170649 |
| "4283" "ENSBTAG00000006487" "RPS9" 1.43917505152002 10.9966446164787 14.6166895252963 0.000131742636715694 0.0120422528671975 |
| "7259" "ENSBTAG00000011025" "BTF3L4" 1.33534151564404 9.41219953572683 14.5596255554856 0.000135793155238956 0.012298624114853 |
| "13011" "ENSBTAG00000019804" "SNRNP25" 1.73402336846769 6.97243085673207 14.5015961691491 0.000140040833329784 0.0125680282421057 |
| "10286" "ENSBTAG00000015582" "HMOX1" 1.89491681893204 8.37706769286837 14.4771262916481 0.000141871863701923 0.0126095618805341 |
| "22270" "ENSBTAG00000046008" "nan" 1.6649139168789 6.33241944700944 14.4614407659157 0.000143058238514974 0.0126095618805341 |
| "12126" "ENSBTAG00000018446" "GCA" -1.48662029022349 8.48516676395363 14.4333634283366 0.000145206868916482 0.0126856832738364 |
| "13859" "ENSBTAG00000021083" "RECQL" -2.44181917970605 4.70298917700115 14.3297693095509 0.000153419467911133 0.0132855876071816 |
| "14671" "ENSBTAG00000023186" "nan" 2.59365559171329 5.54750238636546 14.2851877368896 0.000157096223766507 0.0134856862697649 |
| "8516" "ENSBTAG00000012907" "ODF2L" -1.81387636776785 6.41662362595765 14.2537714186352 0.000159740387370173 0.0135944577941237 |
| "8041" "ENSBTAG00000012177" "SNRPD2" 1.49104808935961 8.62286860959818 14.1118976980802 0.000172250821014806 0.0145079246969541 |
| "7897" "ENSBTAG00000011969" "HSPB1" 1.27539821454207 9.62382886282792 14.0992468846118 0.000173413200389038 0.0145079246969541 |
| "5063" "ENSBTAG00000007650" "SLC38A11" 1.57020124719803 6.9188339809613 14.0490781433178 0.000178101114087907 0.0146811786614792 |
| "24490" "ENSBTAG00000048228" "nan" 1.43117595694511 7.63356679027043 14.0453091619611 0.000178458411606311 0.0146811786614792 |
| "200" "ENSBTAG00000000288" "UPF2" -1.88632628248367 6.3530802502924 14.017265352502 0.000181139722173975 0.0147786060934007 |
| "15454" "ENSBTAG00000026986" "TTN" -3.29930222802813 3.95838645033357 13.9305962702822 0.000189685543620364 0.0152366925387566 |
| "13149" "ENSBTAG00000020004" "ZNF800" -6.67978229371366 2.50755610713793 13.9220755477208 0.000190547336732862 0.0152366925387566 |
| "428" "ENSBTAG00000000630" "LSM14A" 1.32560014342867 8.69909205802351 13.9138333559788 0.000191384711791514 0.0152366925387566 |
| "12979" "ENSBTAG00000019757" "SUDS3" -1.62457472879691 7.12186194596001 13.8900680887502 0.000193819984572055 0.0153071271015626 |
| "10364" "ENSBTAG00000015721" "RAVER1" 2.07498182786566 4.98595808706195 13.7795140796644 0.000205565597814589 0.0161059014414732 |
| "9977" "ENSBTAG00000015127" "SDC4" 1.43457639806915 7.2927410281093 13.7167394019312 0.000212551551585604 0.0165221174586857 |
| "3534" "ENSBTAG00000005311" "POLR3H" -2.10937563354989 5.77073998997775 13.6879062668837 0.000215839974047421 0.0166466579984074 |
| "16942" "ENSBTAG00000033322" "SRP9" -2.32288156915996 5.11017127494885 13.6437260124552 0.000220978475526885 0.0169108489178403 |
| "4721" "ENSBTAG00000007130" "ESF1" -1.43813372566252 7.85250517051046 13.6131268270991 0.000224609480164095 0.0169409633405778 |
| "15490" "ENSBTAG00000027204" "nan" 2.14131823333314 4.75243538746008 13.5770292931022 0.00022897031776303 0.0169409633405778 |
| "11814" "ENSBTAG00000017970" "ZYX" 1.87567503332216 5.274194265649 13.5765604560542 0.00022902751431038 0.0169409633405778 |
| "2385" "ENSBTAG00000003548" "GSTP1" 1.27311731213301 9.44627014234416 13.5598735010838 0.000231072669903238 0.0169409633405778 |
| "9463" "ENSBTAG00000014388" "EIF3I" 1.22421203212598 9.21657879707737 13.5428250274994 0.000233181141349439 0.0169409633405778 |
| "13972" "ENSBTAG00000021250" "RALBP1" -2.17162671563111 5.02596259837397 13.5417305674152 0.000233317159191412 0.0169409633405778 |
| "9619" "ENSBTAG00000014609" "PPP1R12A" -3.09015175897433 4.80991763219843 13.5411895782229 0.000233384422033891 0.0169409633405778 |
| "24405" "ENSBTAG00000048143" "WBP5" -6.63138142474241 2.46309784892873 13.4940611174568 0.000239319682327871 0.0172449919995675 |
| "10613" "ENSBTAG00000016098" "C22H3ORF10" 1.41116030437701 7.29413690558398 13.4666716884833 0.000242838702669842 0.0173717657446137 |
| "14463" "ENSBTAG00000022005" "TLDC2" -6.89454353632988 2.47146255550832 13.4089010900089 0.00025043308439963 0.0177861540229723 |
| "4124" "ENSBTAG00000006225" "RPA2" 1.39051743915803 8.51159097432528 13.3926430988081 0.000252613165985501 0.0178128369614919 |
| "9361" "ENSBTAG00000014226" "RPL34" 1.45378559197384 12.6157335579782 13.3652800403183 0.000256325635193098 0.0178268655361566 |
| "806" "ENSBTAG00000001182" "CDC10" -1.25377497272613 8.51345136720137 13.3539302839825 0.00025788159749594 0.0178268655361566 |
| "17284" "ENSBTAG00000035030" "TMED7" -2.16455476333218 5.64854106443598 13.3514018901506 0.000258229514958508 0.0178268655361566 |
| "15458" "ENSBTAG00000026995" "PNN" -1.49934831450647 8.56829708736391 13.2701777638438 0.000269661634328469 0.0184868031534072 |
| "18319" "ENSBTAG00000038866" "UBE2I" 1.74600781940934 7.5361438243714 13.1979994195796 0.000280248116525701 0.0190800648713222 |
| "18984" "ENSBTAG00000040308" "RPS13" 1.2472215293529 12.5641676535134 13.1052366239909 0.000294471478266903 0.0199111125578826 |
| "11050" "ENSBTAG00000016764" "UBA1" 1.33859417347289 8.39259797117214 13.0759407501671 0.000299112955895968 0.0200873680313265 |
| "6502" "ENSBTAG00000009848" "GPR171" -3.06237103198776 4.10513985922294 13.0098303865829 0.000309860006816452 0.0206684999141352 |
| "12472" "ENSBTAG00000019015" "IFITM3" 1.36642280592083 7.95318220463243 12.9093696700725 0.000326941375448049 0.0216615118014976 |
| "14622" "ENSBTAG00000022902" "RPL17" 1.3790683193251 12.6681547902334 12.8734526999128 0.000333276162277634 0.021934015160032 |
| "7171" "ENSBTAG00000010884" "NKAP" -2.57757101719321 4.77879992790494 12.8210161147798 0.000342747530881489 0.0224079710255765 |
| "16286" "ENSBTAG00000031061" "POLR2L" 1.39570644692901 8.05273450772303 12.7539468866996 0.000355258715813403 0.0230731187007231 |
| "23540" "ENSBTAG00000047278" "NDUFB11" 1.34424569520894 8.18320871676587 12.688222496987 0.000367965893111546 0.0236445790998885 |
| "10387" "ENSBTAG00000015753" "RSBN1L" -2.40589066295062 4.91656383404839 12.6837465368361 0.000368847769589022 0.0236445790998885 |
| "16848" "ENSBTAG00000032954" "ATP5E" 1.27570372534253 10.8117698874005 12.5968398294772 0.000386399511080346 0.0246099095057108 |
| "1601" "ENSBTAG00000002378" "NUCB1" 2.42149851282414 4.74831069020344 12.4953567163987 0.000407964725689345 0.0258168446923411 |
| "524" "ENSBTAG00000000778" "HSP90AB1" -1.5133035682441 11.3055885655377 12.4449756705139 0.000419117745839225 0.0263536967320053 |
| "9183" "ENSBTAG00000013953" "CALD1" -1.51330959147823 8.47040276042306 12.4252655293226 0.000423564311865147 0.0264647271312198 |
| "10556" "ENSBTAG00000016010" "KLHL7" -2.83511528138809 5.53029697151918 12.3716229590609 0.000435908526363928 0.0269768993706811 |
| "12303" "ENSBTAG00000018745" "CEP290" -1.84508578445835 5.71700836724381 12.3659847414114 0.000437226894176356 0.0269768993706811 |
| "7869" "ENSBTAG00000011932" "PRG4" -2.37753539172364 6.6706676298258 12.2642680351309 0.000461714286096509 0.0282916968706582 |
| "11089" "ENSBTAG00000016822" "PPIB" 1.24668537158505 9.15693939878517 12.2539749323344 0.000464268121256749 0.0282916968706582 |
| "18079" "ENSBTAG00000038298" "C22H3ORF19" -2.26469062045085 5.74791333583727 12.1705438430355 0.00048550148587371 0.0294041145309526 |
| "5780" "ENSBTAG00000008736" "CUL1" -2.60986359069459 4.74775844082999 12.136083555965 0.000494555255297046 0.0297698139042222 |
| "12235" "ENSBTAG00000018633" "RABAC1" 1.36259845239659 7.84094906309462 12.1240599505859 0.000497754167026348 0.0297807826477825 |
| "16440" "ENSBTAG00000031598" "GBAS" 1.39043361033642 7.49000351662942 12.0708091147473 0.000512174674204118 0.0304589661671269 |
| "16551" "ENSBTAG00000031871" "NKAPL" -2.98465620708835 4.13219088328855 12.0305550929508 0.000523354881507413 0.0309374813786897 |
| "17350" "ENSBTAG00000035615" "UPF3B" -1.87740263356031 5.52897175780137 12.011349817653 0.00052877547057193 0.0310718538421791 |
| "12411" "ENSBTAG00000018914" "RAB25" 1.81256064216949 5.86765999773503 11.9833804218626 0.000536771229297082 0.0313550625776378 |
| "3227" "ENSBTAG00000004874" "AURKAIP1" 1.43970894493803 6.73223310844755 11.9583417034401 0.000544032679857088 0.031487295817003 |
| "7339" "ENSBTAG00000011145" "NDUFA4" 1.19420325068393 10.3358246513181 11.9536166905608 0.000545414058418508 0.031487295817003 |
| "10680" "ENSBTAG00000016199" "CLINT1" -1.7343806787652 6.66753782946479 11.9197292948941 0.000555425512952643 0.031767151773366 |
| "14044" "ENSBTAG00000021368" "nan" 3.99130820250016 3.28338565905085 11.915468167021 0.000556697453078638 0.031767151773366 |
| "9284" "ENSBTAG00000014103" "SH3GL2" 2.68010629072535 5.03663638851366 11.8972448983302 0.00056217033676679 0.0318950894515043 |
| "7716" "ENSBTAG00000011721" "MED13" -4.1094752108257 2.76068909565662 11.8770957178561 0.000568284881966475 0.0319187990367859 |
| "18136" "ENSBTAG00000038434" "ATRX" -1.29554460194059 7.71096064973923 11.8745742642623 0.000569054764026978 0.0319187990367859 |
| "12516" "ENSBTAG00000019077" "DAD1" 1.2570594498318 9.79983905881785 11.7867796521436 0.000596528377475262 0.0332364873163037 |
| "2204" "ENSBTAG00000003291" "RIMKLB" -1.53453257644708 7.57292334304639 11.7782117449156 0.000599280261578409 0.0332364873163037 |
| "2974" "ENSBTAG00000004471" "ST3GAL6" -1.45633219203097 8.15413438722465 11.7604459111602 0.00060502730170924 0.0332436174533878 |
| "2896" "ENSBTAG00000004348" "MRPL21" 1.51883191371739 6.23458988244151 11.7570143942358 0.000606143754214932 0.0332436174533878 |
| "11500" "ENSBTAG00000017460" "PRORSD1" 1.243280375882 7.8526777989953 11.6920217335835 0.000627686592370932 0.0342349283971593 |
| "6173" "ENSBTAG00000009351" "ATXN10" 1.16780521248577 8.88658018607623 11.6493161411398 0.000642261413329047 0.0348373883098041 |
| "19091" "ENSBTAG00000040551" "ZBTB1" -2.45191165974594 4.25818176781616 11.604060924915 0.000658079662743297 0.0355003411508297 |
| "7705" "ENSBTAG00000011700" "PLLP" 1.47286303422078 7.17576555619677 11.5299563063643 0.000684836236523207 0.0367429528638973 |
| "17121" "ENSBTAG00000034255" "EIF5B" -1.33456298744658 9.16585188298741 11.5078476326371 0.000693029785969966 0.0368864692481546 |
| "11552" "ENSBTAG00000017545" "FCHO2" -4.64788790933539 2.63461298368446 11.5026132392792 0.000694984124813285 0.0368864692481546 |
| "8626" "ENSBTAG00000013063" "ITGB1BP1" 1.28001262723759 7.70237842082743 11.4725275675117 0.000706325516991305 0.0372879438702576 |
| "17068" "ENSBTAG00000033887" "RPL36A-HNRNPH2" 1.65622336518554 6.43107732549051 11.4505273417952 0.000714737134391593 0.0375313031420947 |
| "15354" "ENSBTAG00000026428" "ATP6V0C" 1.16337559243521 9.83095547718437 11.4398005154206 0.00071887508366442 0.0375488615128844 |
| "3347" "ENSBTAG00000005043" "TIMP1" 1.19881920498114 8.11386950111353 11.4283987825339 0.000723299922475262 0.0375811412351357 |
| "3792" "ENSBTAG00000005726" "HNRNPA2B1" -1.16547493035834 9.185370351574 11.3591384507488 0.000750776429901728 0.0388045283559679 |
| "6332" "ENSBTAG00000009580" "SH3BGRL3" 1.49024264606754 8.12128431296515 11.3341431985044 0.00076094934868271 0.0391069253689761 |
| "10323" "ENSBTAG00000015648" "DNAJC21" -1.99353269926942 5.47942357682006 11.3253773318089 0.000764549898319732 0.0391069253689761 |
| "15165" "ENSBTAG00000025434" "ZFP36L1" -1.49634432534505 6.78792828661748 11.2745880424951 0.000785752895640556 0.0399842916791936 |
| "7258" "ENSBTAG00000011024" "TXNDC12" 1.43906133897649 7.69428421826171 11.24695786621 0.000797536224304587 0.0403757825965892 |
| "9280" "ENSBTAG00000014099" "YTHDC2" -2.48162741406938 4.62473399566387 11.2318511706674 0.00080405406063732 0.0404980698296512 |
| "3271" "ENSBTAG00000004934" "NEMF" -2.00015168975843 6.86739872815137 11.2073048417992 0.000814759665777302 0.0408289716779367 |
| "12146" "ENSBTAG00000018479" "PTPMT1" 1.38284589661038 6.67398253704261 11.171832529672 0.000830485575145994 0.0409524411558092 |
| "14035" "ENSBTAG00000021351" "MED12" 1.72446774056169 5.36791055691318 11.1713139318431 0.000830717745780918 0.0409524411558092 |
| "12810" "ENSBTAG00000019502" "MED4" -1.49371917317737 6.65030586531411 11.1683091199119 0.000832064259345134 0.0409524411558092 |
| "7400" "ENSBTAG00000011239" "SEC11A" 1.14695869531177 9.61356803277785 11.1644053153774 0.000833816923857135 0.0409524411558092 |
| "9724" "ENSBTAG00000014766" "SRSF1" -1.31530130275825 8.98901603587248 11.1482105213902 0.000841127728710074 0.0410180434424374 |
| "10773" "ENSBTAG00000016331" "ROGDI" 1.58782667345441 6.37345726213205 11.143068301168 0.000843462603202472 0.0410180434424374 |
| "1389" "ENSBTAG00000002066" "MYL7" 1.47992607085864 7.33407596315371 11.1284028079033 0.000850157659075749 0.0411409627960578 |
| "6893" "ENSBTAG00000010463" "nan" -3.25648792100176 3.61610553573784 11.1121267680826 0.000857650868505293 0.0413011188969963 |
| "10752" "ENSBTAG00000016298" "TM9SF3" -1.40746724875916 7.46049378367586 11.0750672415335 0.000874962469861867 0.0417845127224799 |
| "6253" "ENSBTAG00000009471" "CEP135" -3.09339914817707 3.92312710337947 11.0725434249892 0.000876154186948272 0.0417845127224799 |
| "5726" "ENSBTAG00000008648" "PRDX5" 1.32844897811168 7.91044705430209 11.0544022204948 0.000884768678386298 0.0418563365696343 |
| "6093" "ENSBTAG00000009231" "NSDHL" 1.34746573359082 8.18774477602943 11.051530790339 0.000886140026646431 0.0418563365696343 |
| "16197" "ENSBTAG00000030769" "KPTN" 1.95128714626623 4.62230945869696 11.0286905143756 0.000897124973934365 0.042173417822286 |
| "1749" "ENSBTAG00000002610" "FKBP3" 1.39472553884764 8.87118220775274 10.9852468042446 0.000918400511080016 0.0429689566131845 |
| "12228" "ENSBTAG00000018613" "NOL8" -1.6719102334715 5.87415901711103 10.8761616820926 0.000974099111236047 0.0452883330092899 |
| "335" "ENSBTAG00000000500" "FLJ20565" 1.80528104376954 7.06026401509159 10.8703720133911 0.000977149000301738 0.0452883330092899 |
| "689" "ENSBTAG00000001017" "SLK" -2.32563137837315 4.97109580743234 10.8314269601063 0.000997917011700221 0.0457695606285913 |
| "16826" "ENSBTAG00000032875" "BEX2" -2.45067982027965 4.00837806540702 10.8269832393168 0.00100031488724549 0.0457695606285913 |
| "5800" "ENSBTAG00000008772" "SMC2" -2.00159232509219 7.50872374098055 10.8249001390481 0.00100144095378603 0.0457695606285913 |
| "236" "ENSBTAG00000000347" "RHOG" 2.13624456544189 4.52567524545771 10.791640527332 0.00101959481947061 0.0463845163954554 |
| "3084" "ENSBTAG00000004640" "nan" -6.22227134049883 1.90356665930152 10.7708371718122 0.00103111860652397 0.046688179148347 |
| "960" "ENSBTAG00000001435" "ARL6IP6" 1.40918618664325 6.83280200800177 10.7625810105908 0.0010357284474765 0.046688179148347 |
| "14016" "ENSBTAG00000021325" "SLC19A3" 1.42268615107938 6.76807813584585 10.7467099112203 0.00104464873430987 0.0468762377504867 |
| "3749" "ENSBTAG00000005664" "YWHAE" -1.11832019828658 10.2823803188846 10.6944605449403 0.00107456802678138 0.0480006133954107 |
| "7809" "ENSBTAG00000011849" "HDAC2" -2.1102573166066 5.17206873519432 10.6855835956351 0.00107973666505109 0.0480142358440737 |
| "10837" "ENSBTAG00000016441" "ZNF622" 1.21700113970546 7.7182316772402 10.6533863926695 0.0010986955567727 0.0485228882938279 |
| "5952" "ENSBTAG00000009021" "FEZ2" -2.23208511272121 5.1225317134208 10.6431289901564 0.00110480594756973 0.0485228882938279 |
| "14989" "ENSBTAG00000024701" "TIMM10" 1.5010133260941 6.44666499327405 10.6412637455201 0.00110592077249912 0.0485228882938279 |
| "13049" "ENSBTAG00000019855" "MAP3K15" -2.09857141020242 4.88458373090628 10.6181105441753 0.0011198541557582 0.0489168151577209 |
| "10795" "ENSBTAG00000016363" "TFG" -1.34837167864539 6.91136751698235 10.5850758434644 0.00114004219605408 0.0495792799975588 |
