# Supplementary figures and images for: Male fertility status is associated with DNA methylation signatures in sperm and transcriptomic profiles of bovine preimplantation embryos
Source: BMC Genomics. 2017 Apr 5;18:280. doi: 10.1186/s12864-017-3673-y (PMC5382486; doi:10.1186/s12864-017-3673-y)

## DMRs by Chromosomes

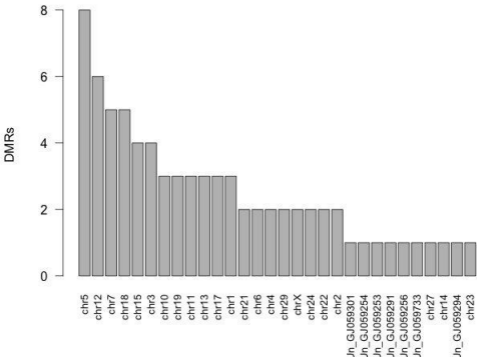

Supplement: Supplementary file 5 — Distribution of the DMRs across chromosomes. The histogram represents the number of DMRs located on each chromosome. unk: represents regions with an unknown location as they do not map to a chromosome. (PDF 34 kb) [file 12864_2017_3673_MOESM5_ESM.pdf]

PCA: Condition

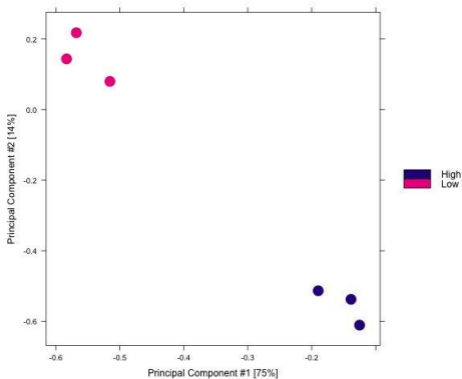

Supplement: Supplementary file 7 — Principal component analysis for high and low fertility pools. (PDF 21 kb) [file 12864_2017_3673_MOESM7_ESM.pdf]
